# Residual Shrinkage ViT with Discriminative Rebalancing Strategy for Small and Imbalanced Fault Diagnosis

**DOI:** 10.3390/s24030890

**Published:** 2024-01-30

**Authors:** Li Zhang, Shixing Gu, Hao Luo, Linlin Ding, Yang Guo

**Affiliations:** College of Information, Liaoning University, Shenyang 110036, China

**Keywords:** fault diagnosis, classimbalanced data, small sample, vision transformer, deep residual shrinkage networks, loss function

## Abstract

In response to the challenge of small and imbalanced Datasets, where the total Sample size is limited and healthy Samples significantly outweigh faulty ones, we propose a diagnostic framework designed to tackle Class imbalance, denoted as the Dual-Stream Adaptive Deep Residual Shrinkage Vision Transformer with Interclass–Intraclass Rebalancing Loss (DSADRSViT-IIRL). Firstly, to address the issue of limited Sample quantity, we incorporated the Dual-Stream Adaptive Deep Residual Shrinkage Block (DSA-DRSB) into the Vision Transformer (ViT) architecture, creating a DSA-DRSB that adaptively removes redundant signal information based on the input data characteristics. This enhancement enables the model to focus on the Global receptive field while capturing crucial local fault discrimination features from the extremely limited Samples. Furthermore, to tackle the problem of a significant Class imbalance in long-tailed Datasets, we designed an Interclass–Intraclass Rebalancing Loss (IIRL), which decouples the contributions of the Intraclass and Interclass Samples during training, thus promoting the stable convergence of the model. Finally, we conducted experiments on the Laboratory and CWRU bearing Datasets, validating the superiority of the DSADRSViT-IIRL algorithm in handling Class imbalance within mixed-load Datasets.

## 1. Introduction

Mechanical rotating components play a pivotal role in various domains of modern industry, encompassing engines, motors, turbines, and more [1,2,3]. Among these components, bearings, serving as the essential supporting elements of mechanical rotating parts, bear the crucial functions of load transmission and friction mitigation. However, when operating for extended periods under complex conditions, mechanical rotating components often experience an array of failures, such as bearing Rolling element wear, loose cages, and inner Ring fracture, among other issues [4,5]. These failures not only lead to interruptions in industrial production, but also pose the risk of severe consequences, including equipment damage, accidents, and even personnel injuries. Therefore, accurate and timely monitoring and diagnosis are critical in ensuring equipment reliability and maintaining production efficiency [6].

In real industrial production, machines operate in a healthy state for the majority of their operational time, and the probabilities of failures in rotating mechanical components vary. Moreover, it is crucial to note that the diagnostic learning direction of deep learning (DL) models tends to shift towards categories with abundant Samples; thus, neglecting the minority Classes that it cannot adequately grasp, this phenomenon results in model overfitting [7], ultimately impacting the diagnostic efficiency of model Classification and increasing equipment maintenance costs. Hence, the exploration of fault diagnosis in the context of rotating mechanical components, while dealing with the constraints of small and imbalanced data, carries profound practical significance [8,9].

The primary objective of feature-based methods is to enhance the model’s ability to extract features, particularly the capacity to extract critical information from limited Samples. The introduction of attention mechanisms has alleviated this issue to some extent [10,11], but existing attention-based methods may lack modeling of internal relationships within signal sequences. The Vision Transformer (ViT) architecture [12], which has robust Global feature extraction capabilities by modeling internal data information, has gained favor among many researchers. ViT models can be categorized into pure attention-based ViT models and convolutional ViT models, depending on whether convolutional operations are incorporated into the ViT architecture. In the former category, ViT mainly relies on the self-attention mechanism, enabling the model to establish long-distance dependencies between different locations, which helps to recognize complex patterns and correlations and, thus, diagnose faults more accurately [13]. In addition, some researchers have used ViT to achieve long-distance-dependent modeling of signals in complex environments in real industrial production. Zhou et al. [14] proposed an Industrial Process Optimization ViT (IPO-ViT) method, which significantly enhances the robustness of the model by exploring the Global receptive field. Exclusively attention-driven ViT models, as mentioned above, often lack further precise analysis of local features in vibration signals, leading to essential features being submerged in redundant information.

In modeling local features and filtering out redundant information from the signal, filters play a pivotal role in this process. Convolutional Neural Networks (CNNs) excel at capturing the local details of an image, while ViT excels at understanding the Global context of an image. This multimodal fusion model provides a more-comprehensive understanding of the image content, which improves the Accuracy of recognition and Classification. Therefore, attention mechanisms must be combined with CNNs to address their limitations in computer vision [15,16]. Moreover, the incorporation of the Wavelet transform into the Transformer framework has been undertaken to attenuate persistent noise from the signal. Tian et al. [17] introduced a Wavelet-based self-attention Network, which utilizes self-attention mechanisms through multiple frequency-oriented fusion modules to extract signal features hidden in interference information. Furthermore, considering the complexity of equipment operating environments, mixed-load complex data are more aligned with modern industrial production settings. The Deep Residual Shrinkage Network (DRSN) has been proven to have the ability to model features in complex data in the field of fault diagnosis. Pei et al. [18] proposed an improved Deep Residual Shrinkage Network that can judge whether bearing performance is deteriorating based on the ratio of the fault features to the noise capability. Some scholars have combined the DRSN and Transformer to improve the fault-detection performance of target machines. Chen et al. [19] introduced a new Transformer-based Deep Residual Shrinkage Network, which eliminates potential interference features in the input signal through the DRSN and, then, feeds them into an enhanced Transformer model to determine their category through Sample comparison.

Classifier-based strategies, aimed at improving model recognition rates, involve the use of Loss functions to assign different weights to Classes, thereby ensuring the efficient extraction of relevant information from Samples across various categories. However, in the case of imbalanced Datasets, it is discriminative features, and it is indeed challenging to capture highly discriminative features, particularly for fault categories with scarce representation. In such scenarios, the characteristics of certain fault types may overlap significantly with others, complicating the model’s ability to discern distinct patterns. These ambiguous Samples, which we term “difficult Samples”, pose a substantial challenge to the effectiveness of the Classification model. Therefore, a series of cost functions have been proposed, such as the Label-Distribution-Aware Margin Loss (LDAML) [20], Class-Balanced Loss (CB) [21], Focal Loss (FL) [22], etc., In the field of fault diagnosis, Zhao et al. [23] introduced a novel approach that combines Focal Loss with random oversampling to address Class imbalance in deep Neural Networks. Xiao et al. [24] combined cost learning with a symmetric regularization criterion and proposed a Selective Deep Ensemble model based on the Group Method of Data Handling (GMDH) technology for cost-sensitive selective Deep Ensemble prediction. These functions often exhibit sensitivity to the proportions of different Classes, overlooking the presence of difficult Samples within Classes. Moreover, in the face of severe imbalance with extremely limited small Samples, the features of Intraclass difficult Samples cannot be fully exploited, further restraining the superiority of cost-sensitive cost functions.

In summary, the limitations of the above methods in addressing the challenges of small and imbalanced cases can be attributed to two main reasons: Regarding the issue of small Samples, while attention mechanisms can help Networks focus on essential parts of the input signals, they tend to overlook the complex associations and dependencies between different elements within the signals. This limitation makes it challenging to learn the dependency relationships of Global features in the signals, resulting in some attention being erroneously directed toward local redundant features. Concerning the problem of Class imbalance, the currently proposed Class imbalanced cost functions often prioritize the quantity proportions of different Class Samples. However, they tend to overlook the persistence of Intraclass difficult Samples, particularly when dealing with severely imbalanced Datasets. This oversight can lead to model overfitting.

To address the first issue, we introduce a Neural Network module designed to selectively eliminate redundant signal data in an adaptive manner. This module, referred to as the Dual-Stream Adaptive Deep Residual Shrinkage Block (DSA-DRSB), is proposed. Specifically, to mitigate the suboptimal performance of one-dimensional data in the CNN [25], the raw vibration signals are transformed into two-dimensional time–frequency maps using the Continuous Wavelet Transform (CWT) to capture fine temporal resolution and frequency changes. Furthermore, to tackle the challenges posed by small Sample Datasets, the DSA-DRSB is introduced. More specifically, we improved and merged two different Deep Residual Shrinkage Blocks. One Branch, with a threshold fusion mechanism, excels at eliminating redundant information within the data, while the other Branch outperforms in extracting detailed fault features. The extracted features from both Branches are then fused, enabling the Network to dynamically adjust its output based on the adaptive input data under conditions of extremely limited Samples.

Addressing the second issue, which involves the challenge of adapting to severely imbalanced Datasets, a novel Loss function is introduced, referred to as Interclass–Intraclass Rebalancing Loss (IIRL). While existing imbalance-Aware Loss functions have been extensively researched, they primarily focus on adjusting the contributions between different Classes and do not fully exploit the crucial features of individual difficult Samples within Classes. IIRL introduces online Hard Sample mining techniques within CB Loss. Specifically, for challenging Samples, the top n most-difficult Samples are selected for in-depth mining of potential fault information. Ultimately, during model training, IIRL decouples the contributions of Interclass and Intraclass Samples, thereby enabling the deep exploration of Sample-specific fault features. The primary contributions of this work are as follows:

1. A method is proposed for small and imbalanced intelligent fault diagnosis, namely DSADRSViT-IIRL. This method exhibits high diagnostic Accuracy and strong robustness in scenarios with extremely limited Samples and severe Class imbalance cases, whose application prospect is promising.
2. A new DSA-DRSB Branch with threshold fusion is designed. The pathway with threshold fusion adeptly captures locally key-sensitive feature vectors, which have a significant impact on the outcome, while skillfully modeling Global features. In parallel, the other pathway employs a shared-threshold strategy to alleviate the impact of redundant information, enabling the model to capture critical fault discrimination features from exceedingly limited Samples.
3. A novel Loss function, IIRL, is proposed to address Class imbalance issues. This is achieved by introducing online Hard Sample mining techniques into the Class imbalanced cost function, aiming to tackle the persistence of difficult Samples within the same category. It allows for a thorough exploration of challenging Intraclass Samples, to decouple the contributions of Interclass and Intraclass Samples during model training. This is especially crucial in scenarios characterized by seriously imbalanced and extremely limited Sample sizes.
4. The experimental results on the CWRU bearing Dataset and the Laboratory bearing Datasets, conducted under complex operating conditions, demonstrate that the model exhibits good generalization abilities compared to the most-recent relevant algorithms.

The remainder of the paper is organized as follows: Section 2 briefly introduces the fundamental theory of fault diagnosis models. The DSADRSViT-IIRL, DSA-DRSB, and IIRL are detailed in Section 3. Section 4 validates the effectiveness of the proposed method using the CWRU and Laboratory bearing Datasets. Finally, conclusions are drawn in Section 5.

## 2. Background and Related Works

### 2.1. The Vision Transformer

**Figure 1:**
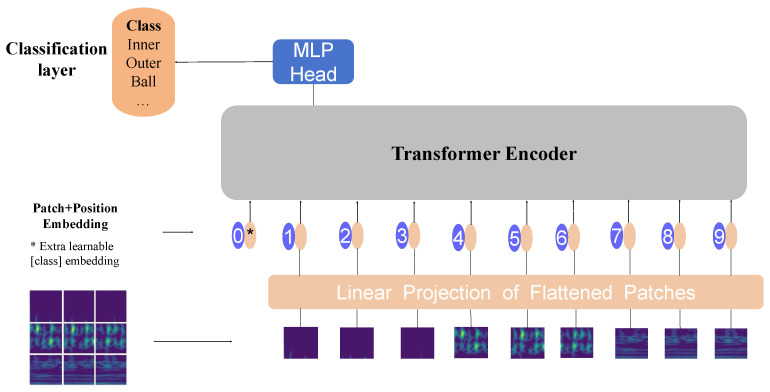
The architecture of ViT.

ViT [12] was developed as an extension of the Transformer architecture for various mainstream computer vision tasks. In image Classification, ViT differs from the CNN in how it processes information. Instead of using stacked convolutional layers to extract features, ViT utilizes self-attention mechanisms, enabling it to focus more efficiently on crucial regions within the image. The ViT model consists of Patch Embedding, the Transformer Encoder, and a final Classification layer, as illustrated in Figure 1.

The Embedding layer is responsible for converting the image into a 1D token representation that can be processed by the Transformer model. It divides the input $x\in R^{H\times W\times C}$ into *N* equally sized non-overlapping patches, where $N=HW/P^{2}$, and then, it embeds the positional information for each pixel within the patches. The transformation process is described by Equation (1).

(1) $$Z=\left[x^{cls};x_{1}^{p}E;x_{2}^{p}E;\cdots ;x_{n}^{p}E\right]+E^{pos},E_{pos}=R^{(N+1)\cdot D}$$

where *E* is a shared trainable patch embedding space matrix, *E* $\in R^{p^{2\times C\times D}}$, $x^{cls}$ is a learnable vector, *D* represents the dimensionality of the vectors, and $E_{pos}$ is a learnable positional information matrix.

The Transformer Encoder layer consists of L identical sub-layers, with each sub-layer comprising four components: Multi-Head Self-Attention (MSA), Multi-Layer Perceptron (MLP), Layer Normalization (LN), and Residual Connection (RC). One of the core components of ViT, MSA, is used to capture Global correlations and contextual information within the input feature vector sequence. It performs more-complex mappings on the features at each position to enhance the model’s representational capacity. LN is employed to normalize the features at each position, ensuring that the inputs between different positions have similar distributions, which aids in better learning. RC adds the input to the output, allowing information to flow more easily through the Network layers. This helps mitigate the problem of gradient vanishing. The specific propagation process is represented by Equations (2) and (3).

(2) $$Z_{k}^{\prime }=MSA\left(LN\left(Z_{k-1}\right)\right)+Z_{k-1},k=1,\ldots ,L$$

(3) $$Z_{k}=MLP\left(LN\left(Z_{k}^{\prime }\right)\right)+Z_{k}^{\prime }$$

where $Z_{k-1}$ denotes the result after Patch Embedding and $Z_{k}$ denotes the final output of the Transformer Encoder. It can be expressed by Equation (4) as follows:

(4) $$Z_{k}=\left[Z_{k}^{cls};Z_{k}^{1};,Z_{k}^{2};\cdots ;Z_{k}^{N}\right]$$

ViT uses the output of the last Transformer Encoder block as the Global feature representation of the image and feeds it into the final classifier to obtain the Classification result, as shown in Equation (5).

(5) $$y=\vartheta (LN\left(Z_{k}^{cls}\right))$$

where ϑ represents the linear layer.

### 2.2. Deep Residual Shrinkage Network

The Deep Residual Shrinkage Network (DRSN) is a novel Network Classification model for the diagnosis of rotating machinery faults [26]. The DRSN comprises convolutional layers, one or more Residual Shrinkage Blocks (RSBUs), batch Normalization layers, activation functions, Global Average Pooling (GAP), and fully connected layers. The DRSN is designed to effectively capture and classify fault patterns in rotating machinery, making it a valuable tool in fault diagnosis applications.

**Figure 2:**
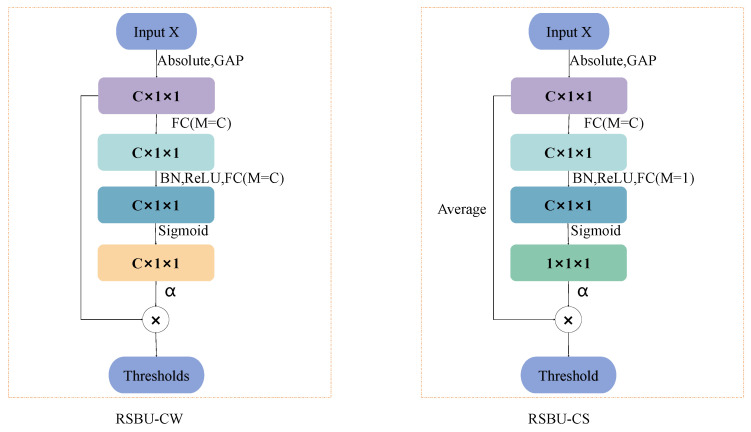
DRSN-CW and DRSN-CS.

The RSBU, as a core component of the DRSN, is tasked with obtaining one or a set of soft thresholds. The only difference between the DRSN with channelwise thresholds (DRSN-CW) and the DRSN with channel-shared thresholds (DRSN-CS) lies in how the Average is computed within the Residual Shrinkage Block (RSBU), as shown in Figure 2. In the DRSN-CW, the RSBU calculates a separate threshold for each channel (while the DRSN-CS uses a channel-shared threshold), enabling adaptive thresholding. To select the most-significant features in the image, reduce the data dimensionality, and enhance the model’s interpretability, a soft thresholding function is used to accomplish these tasks. The process can be represented by Equation (6).

(6) $$f\left(x\right)=\left\{\begin{array}{cc} x_{c}-\gamma_{c}, & if\;x_{c}>\gamma_{c},\\ 0, & if\;-\gamma_{c}\leq x_{c}\leq \gamma_{c},\\ x_{c}+\gamma_{c}, & if\;x_{c}<-\gamma_{c} \end{array}\right.$$

where $y_{c}$ denotes the output feature of the *c*th channel. Since the amplitude of the noise feature information in the Network is usually small, the soft threshold function returns the features with thresholds between $\left[-y_{c},y_{c}\right]$ and 0, while retaining the feature values larger than $y_{c}$ and smaller than $-y_{c}$. The sparse representation of the signal features is captured by eliminating the noise and redundancy information to achieve the purpose of data dimensionality reduction, and in this way, a more-simplified and -efficient signal processing model is obtained.

## 3. The Proposed Method

### 3.1. Proposal of the Dual-Stream Adaptive Deep Residual Shrinkage Block

A novel Neural Network block called the DSA-DRSB is introduced, which can adaptively adjust the outputs of two sub-Networks based on the redundant information and fine-grained features within the input Dataset, all without requiring additional expert prior knowledge.

Although ViT has shown satisfactory results in numerous computer vision tasks, the CNN is more sensitive to local structures when dealing with 2D images. The CNN inherently possesses certain properties such as a degree of shift, scale, and distortion invariance [27].

**Figure 3:**
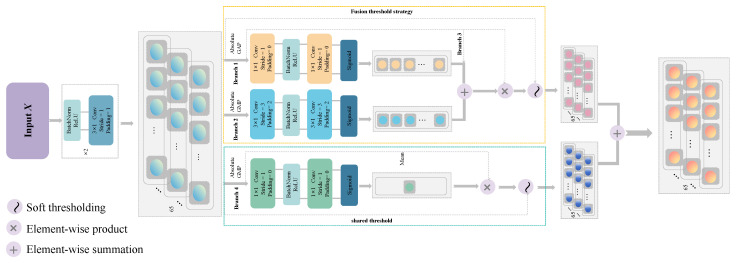
The architecture of DSA-DRSB.

Firstly, to address the issue of the CNN outperforming ViT models of similar scale on small training sets [28], as shown in Figure 3, we embedded the proposed DSA-DRSB after the Patch Embedding of ViT, aiming to embed the inherent desirable attributes of the CNN into ViT. This ensures the capturing of even subtle key feature vectors in each token. Therefore, the output of Patch Embedding is first fed into the DSA-DRSB, where 3 × 1 filters are used for feature extraction. For each token that retains both positional information and signal features after segmentation, smaller kernel convolutions with a 3 × 1 size provide a smaller receptive field. This is crucial for discerning the differences and patterns in fine structures among different tokens. Additionally, to prevent gradient explosion during Network training, batch Normalization (BN) is performed before the convolution operation to stabilize the Network’s input. Furthermore, to enhance the model’s robustness and generalization capability, a non-linear activation function, ReLU, is introduced after each convolution to enhance the Network’s non-linear representation capability. The specific process is illustrated in Equation (7).

(7) $$M=\sum_{i=0}^{C}\sigma \left(BN\left(x_{i}\right)\right)*w_{i}j+b_{j}$$

where $x_{i}$ represents the feature map, $w_{i}j$ and $b_{j}$ are the weight and offset matrices, respectively, BN(·) is the batch Normalization, and is the nonlinear activation function.

Secondly, Average Pooling runs the risk of smoothing local critical bands when focusing on Global information [29]. To get out of the dilemma that the traditional DRSN-CW (Branch 1) uses Global Average Pooling as the only criterion for threshold acquisition, we no longer deem the Average channel feature vector obtained solely through Global Average Pooling (GAP) as paramount (Branch 1). Instead, we propose a novel fusion threshold module—the DSA-DRSB—as illustrated in Figure 3. In the proposed approach, we introduce two supplementary threshold acquisition Branches: one Branch, obtained through Global maximum Pooling (GMP), captures the maximum feature vector in Pooling (Branch 2), while another Branch focuses on the original feature vectors (Branch 3). The maximum features contribute by responding to the local key frequency bands with the highest gradients in the original signal [30], and the contribution of the original features ensures that threshold learning does not escalate with the increase in the Network layers [31]. Specifically, Branch 1 and Branch 2 utilize the Sigmoid function after two convolutional layers to scale the thresholds for each channel into the (0, 1) range. Employing convolutional layers instead of linear layers for threshold acquisition effectively reduces the computational burden and enhances the model’s generalization capacity, as detailed in Equations (8) and (9), respectively.

(8) $$\gamma_{avg_{c}}=\frac{1}{1+e^{-M_{avg_{c}}}}$$

(9) $$\gamma_{max_{c}}=\frac{1}{1+e^{-M_{max_{c}}}}$$

where $M_{avg_{c}}$, $M_{max_{c}}$ represent the *c*th channel of the feature map after Average Pooling and maximum Pooling, respectively, and similarly, γ represents the attention score of the *c*th channel of the feature map. Following $M_{avg_{c}}$, $M_{max_{c}}$, the fusion threshold strategy now enables the precise capturing of crucial critical bands in the time–frequency spectrogram while maintaining control over Global feature vectors, thereby reducing the filtering costs.

Then, the obtained adaptive attention, as well as the original data are fused, and the fusion process, as well as the final threshold can be shown in Equations (10) and (11), respectively.

(10) $$\gamma_{c}=\gamma_{avgc}⊕\gamma_{maxc}⊕BN\left(M_{c}\right)$$

where ⊕ denotes the channel-by-channel accumulation process and $M_{c}$ denotes the *c*th channel of the feature map after Average Pooling or maximum Pooling.

(11) $$\tau_{1}=\gamma_{c}\cdot Average\left(M_{i,j,c}\right)$$

where τ represents the final adaptive threshold of the *c*th channel of the feature map *M*.

Similarly, Branch 4, in order to eliminate the influence of random noise and redundant information, applies only the operation of Average attention in the threshold processing. A shared threshold is obtained for each channel, as shown in the specific process in Equations (12) and (13).

(12) $$\gamma_{r}=\frac{1}{1+e^{-M}}$$

(13) $$\tau_{2}=\gamma_{r}\cdot Max\left(M_{\left(i,j,c\right)}\right)$$

where $\gamma_{r}$ and $\tau_{2}$ denote the attention score and threshold size of the feature map *M*, respectively. After traversing this path, the redundant information in individual channel features is further suppressed due to the shared threshold assistance, mitigating the sensitivity of the Network model to interference information.

Furthermore, the combination of channelwise and shared thresholds creates a more “fitting” Network structure and a new way of connection [32]. In the context of image Classification, the channelwise representation capacity is greater than that of the shared threshold connection. However, the combination of channelwise and shared thresholds is even stronger than the two strong structures combined. The outputs of both the left and right Branches can be derived from the DRSN formula. Compared to CNNs, which have a “black-box” nature, this approach offers stronger interpretability. Moreover, to reduce the Loss caused by gradient backpropagation and maximize the preservation of the original signal features, we applied a shortcut operation to each Branch. This helps avoid the problem of gradient vanishing during the Network’s update process.

### 3.2. Proposal of the Interclass–Intraclass Rebalancing Loss

A new Class-Balanced Loss function is introduced, which can adaptively consider Interclass Class Balance and perform online Hard Sample mining based on the characteristics of the input Dataset.

In order to Balance the differences in the number of training Samples across different Classes, the CB Loss Balances Sample quantities by introducing Class weights. The goal of the CB Loss is to make the model pay more attention to minority Classes to improve overall Classification performance, as shown in Equation (14).

(14) $$CB_{sigmoid}\left(z,y\right)=-\frac{1-\beta }{1-\beta^{n_{y}}}\sum_{i=1}^{C}log\left(\frac{1}{1+exp(-z_{i}^{t})}\right)$$

where β=(N−1)/N is the number of Samples, *N* is the number of Samples in each category, $n_{y}\left(ground-truth\right)$ is the number of Samples in a batch, *C* is the model prediction, $z_{i}^{t}$ is the model input, and *z* and *y* are the labeled values.

However, the CB Loss has some limitations and drawbacks in practical applications, which restrict its effectiveness and applicability in specific scenarios. One of its main limitations is that it only adds extra attention to minority Classes, without fully considering the possibility of Hard Samples within the same Class. This issue becomes particularly prominent when dealing with extremely limited and imbalanced data.

To address this problem, we propose a new method called Interclass–Intraclass Rebalancing Loss (IIRL). This method consists of two parts: The first component employs Class-adaptive weights, determined using the CB Loss, to regulate the attention levels for different Classes. The second part is the Hard Sample Mining Loss (HSML) function, which performs online Hard Sample sorting based on the feedback values obtained from the Neural Network during backpropagation, as shown in Equation (15).

(15) $$HSML\left(x,y\right)=DES\sum_{i=1}^{C}y_{i}log\left(x_{i}\right)$$

where *x* is the model input, *y* is the labeled value, *C* denotes the number of categories in the Sample, and DES denotes the descending order in which the resulting Loss values are ranked from largest to smallest.

Specifically, we chose to retain the top n Hard Samples. The value of n can be adjusted as needed to ensure that we retain a sufficient number of Hard Samples. If the number of Hard Samples to be retained is smaller than the total number of Samples with high Loss in the current batch, we will select and keep those Samples. Otherwise, we will retain all the Hard Samples. This approach helps in selecting and preserving the most-challenging Samples, which can be crucial for model training, especially in scenarios with limited data and Class imbalance. Therefore, the final Loss of the IIRL is shown in Equation (16).

(16) $$\begin{array}{cc} IIRL= & \left\{\begin{array}{cc} CB_{\mathrm{sigmoid}}\left(z,y\right)+HSML\left(x,y,n,x_{\mathrm{num}}\right), & n<x_{\mathrm{num}}\\ CB_{\mathrm{sigmoid}}\left(z,y\right)+HSML\left(x,y,n\right), & else \end{array}\right. \end{array}$$

where *x* represents the input data, $x_{\mathrm{num}}$ denotes the Sample size of a batch, *n* represents the number of Samples to be retained, *z* is the raw data, and *y* is the Sample labels. The process of obtaining Intraclass challenging gradients for Samples of the same category in the HSML can be represented as:

(17) $$\frac{d(HSML)}{dx}=DES\sum_{i=1}^{n}\frac{y_{i}}{x_{i}}$$

By re-mining the difficult Samples within the same category, at this time, the contribution of Interclass and Intraclass Samples to the model is truly discriminative. Additionally, the addition of the gradients of the difficult Intraclass Samples to the total gradient, through the Continuous injection of new gradients, further inhibits the occurrence of the model overfitting phenomenon and improves the model’s generalizability.

Indeed, by distinguishing the importance of different Classes and re-mining deep features from Hard Samples within the same Class, the proposed Interclass–Intraclass Rebalancing Loss (IIRL) method helps in making the decision boundaries between different Classes clearer. It allows for more-effective utilization of limited Samples to capture crucial, Hard-to-decipher features. Therefore, by decoupling the impact of different Samples between and within Classes on model convergence, this method is suitable for intelligent fault diagnosis tasks in scenarios with Class imbalance. It helps address the challenges posed by imbalanced Datasets and limited Samples in intelligent fault diagnosis.

### 3.3. The Proposed Framework Based on DSADRSViT-IIRL Algorithm

**Figure 4:**
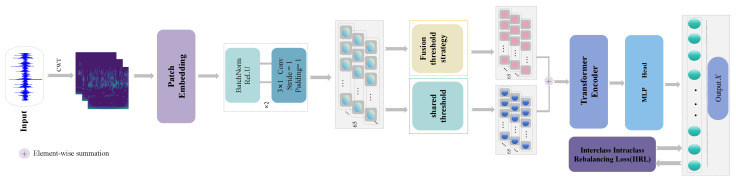
The architecture of DSADRSViT-IIRL.

**Figure 5:**
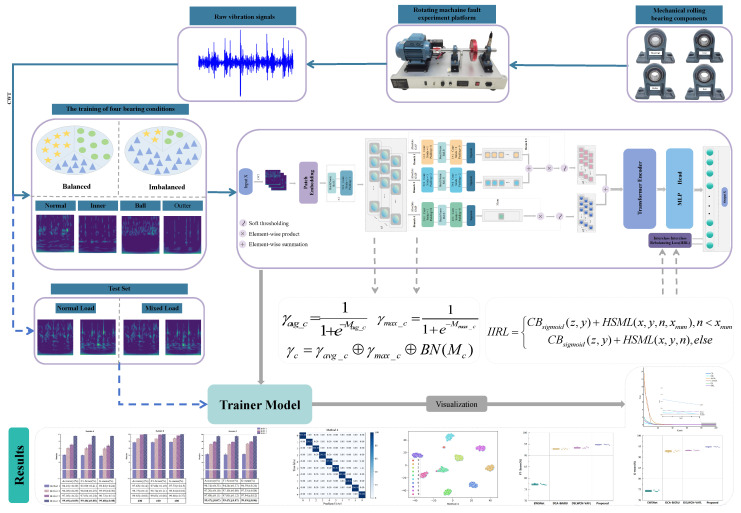
Pipeline of DSADRSViT-IIRL Fault diagnosis method.

In the field of rotating machinery fault diagnosis, the ViT model has gained widespread attention and research interest. However, when compared to CNN-based models, ViT models tend to underperform on small and imbalanced Datasets [33]. In modern industrial precision production processes, the components of rotating machinery often operate within controlled parameters, as even minor faults can trigger significant consequences. Consequently, there is an extremely limited number of labeled fault Samples available. To better address the challenges posed by small and imbalanced Datasets in the context of modern industrial production environments, we propose a fault diagnosis method named DSADRSViT-IIRL, tailored for highly limited Samples and severely imbalanced data, as shown in Figure 4. In practical industrial applications, the fault diagnosis process based on DSADRSViT-IIRL is illustrated in Figure 5, and the specific steps are as follows:

Step 1 Obtain raw one-dimensional vibration signals, and transform them into two-dimensional time–frequency spectrograms using the Continuous Wavelet Transform (CWT).
Step 2 Divide the data into training and testing sets according to a certain ratio.
Step 3 Input the training set into the DSADRSViT-IIRL model, and freeze the trained model parameters.
Step 4 Input the testing set into the model, and obtain the final diagnostic results.

## 4. Experiments and Analysis of Results

### 4.1. Implementation Details

To validate the performance of the proposed imbalanced fault diagnosis method based on the DSADRSViT-IIRL, various experiments were conducted on both the Case Western Reserve University (CWRU) bearing data and the Laboratory bearing data. All experiments were implemented in PyTorch 1.10.2+cu113, Python 3.9, and run on an AMD Ryzen 7 5800H with a Radeon Graphics @3.20 GHz, GTX 3050TI GPU.

When dealing with class imbalanced data for fault diagnosis, even if Samples from the major Class are misclassified, the model’s Accuracy may still appear relatively high. Therefore, relying solely on Accuracy may not be sufficient to comprehensively evaluate the experimental results. In addition to Accuracy, we also introduced the F1-Score and G-mean evaluation metrics to provide a more-comprehensive assessment of Classification performance, as shown in Equations (18)–(20). These metrics better consider the model’s performance on minority Classes and offer more-accurate experimental evaluations.

(18) $$Accuracy=\frac{TP+TN}{TP+FN+FP+TN}$$

(19) $$F1-Score=\frac{2TP}{2TP+FP+FN}$$

(20) $$G-mean=\sqrt{\frac{TP\times TN}{\left(TN+FP)(TP+FN\right)}}$$

where True Positive (TP) is the result of the model correctly predicting the Positive Class. False Positive (FP) are the results when the model incorrectly predicts the Positive category. True Negative (TN) is the result that the model correctly predicts the Negative category. False Negative (FN) is the result of the model incorrectly predicting the Negative category.

In addition, to ensure the fairness of the comparison between the different methods, some specific training parameters are set as shown in Table 1.

### 4.2. Bearing Dataset from CWRU

#### 4.2.1. Data Descriptions

This Dataset was collected and published by researchers in the Department of Mechanical and Aerospace Engineering at CWRU and is primarily used for bearing failure detection and diagnostic studies. The vibration signals were collected by accelerometers at four different loads (0–3 HP) from the drive and fan ends, and each fault corresponds to three damage diameters: 0.18 mm, 0.36 mm, and 0.54 mm, with HP = 2 (1750 rpm), which represent Slight Inner Race fault (SIR), Slight Rolling Ball fault (SRB), Slight Outer Race fault (SOR), Middle Inner Race fault (MIR), Middle Rolling Ball Race fault (MRB), Middle Outer Race fault (MOR), Critical Inner Race fault (CIR), Critical Outer Race fault (COR), and Critical Rolling Ball fault (CRB). As a result, the Dataset comprises one healthy condition and nine fault conditions. Additionally, in the experiments, the original one-dimensional vibration signals were subjected to the Continuous Wavelet Transform (CWT) for decomposition and transformation, resulting in two-dimensional time–frequency feature maps. This transformation was carried out to capture rich time–frequency domain features in the original signal. The CWT results for each fault category are detailed in Table 2. In the literature [34], Zhang proposed that, when the number of training Samples is less than 100, it can be referred to as a small Sample problem, and when the number of Samples is less than 10, it is termed as an extremely small Sample problem. Furthermore, during the Dataset organization process, adjusting the number of Samples in each category to the desired quantity proves to be a highly challenging task.

#### 4.2.2. Partition of Datasets

Similarly, in the context of imbalanced Classes, when $N_{fault}/N_{Normal}\leq$ 0.1, it is referred to as an extremely imbalanced problem [35], where $N_{fault}$ represents the number of fault Samples and $N_{Normal}$ represents the number of Normal Samples. To thoroughly assess the proposed model’s performance on small and imbalanced Datasets, four different Datasets (A, B, C, and D) were created, with the details provided in Table 3. Dataset A has an extremely limited number of training Samples, with only nine available for each Class. Dataset B has a limited number of Samples and is imbalanced, while Dataset C has an extremely limited number of Samples and is highly imbalanced. To assess the model’s ability to fit complex data under the conditions of extremely limited and highly imbalanced Samples, Dataset D is a mixed Dataset containing four different loads, with each load having an appropriate proportion of data in a cross-load mixed Dataset.

#### 4.2.3. Ablation Experiment

**Figure 6:**
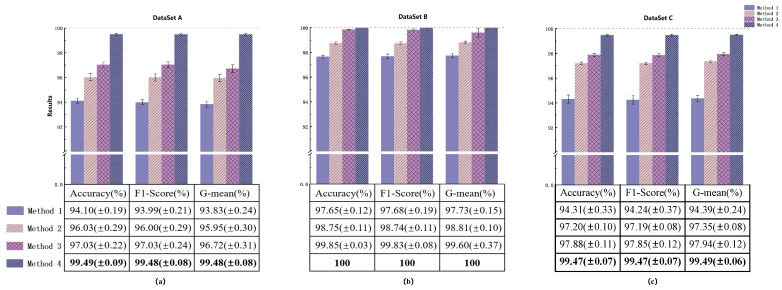
Diagnostic results of various methods in Dataset A (**a**), Dataset B (**b**), and Dataset C (**c**).

In order to verify the performance of the DSADRSViT-IIRL on small and imbalanced Datasets, we conducted ablation experiments on four Datasets, A, B, C, and D. The DSADRSViT-IIRL (Method 4) was compared with other models, namely ViT-CE (Method 1, without the DSA-DRSB and IIRL), ViT-IIRL (Method 2, without the DSA-DRSB), and DSADRSViT-CB Loss (Method 3, without the IIRL). After 100 iterations, the diagnostic diagram of the model is shown in Figure 6. When dealing with the Balanced Dataset, as shown in Figure 6a, by comparing Method 1 and Method 2, in the absence of the DSA-DRSB module, which has been introduced to address issues related to small Sample sizes, we observed that the CE treats each of the data in the same way and cannot give timely attention to the difficult Samples hidden between the Samples, and the anti-interference ability is not satisfactory, which leads to the model ignoring a few difficult Samples; thus, the overfitting phenomenon is found. In contrast, for Method 2, continuously injecting new gradients into the model through the technique of online mining of difficult Samples further suppresses the occurrence of the model overfitting. This results in the Accuracy, F1-Score, and G-mean reaching 96.03%, 96.00%, and 95.95%, respectively, which is a significant improvement over Method 1. Hence, in the absence of deep feature extraction, the IIRL undoubtedly provides a reliable method for solving the problem of Balanced Datasets.

In dealing with imbalanced Datasets, as shown in Figure 6b, especially under extremely imbalanced conditions, as shown in and Figure 6c, as the proportion of imbalanced data gradually becomes more pronounced, a comparison between Method 3 and Method 4 in Figure 6b,c reveals that Method 3, lacking the IIRL, experiences a significant decrease in the Accuracy, F1-Score, and G-mean. It becomes evident that the CB assigns distinct category weights to each data category by applying varying levels of attention. Nevertheless, challenging Samples within categories persist [36], and challenging Samples that are Hard to learn are often falsely assumed to be comprehended by the Neural Network, leading to overfitting. Conversely, the scores of Method 4 can still maintain around 99.47%, once again highlighting that the diagnostic performance of ViT under imbalanced conditions is improved by the online analysis of difficult Samples with the support of the IIRL. By comparing Method 4 with Method 2, you can observe that ViT lacks the feature modeling ability of key time–frequency bands in complex time–frequency diagrams, and the existence of the self-attention mechanism makes its Global sensing field enhanced, while the perception of local details is relatively weak. It is unusually sensitive to the abnormal time–frequency bands in the time–frequency diagrams, resulting in a model that is not as robust as it should be. The DSA-DRSB maximizes the utilization of crucial time–frequency regions by progressively identifying local critical information and modeling Global information through the threshold fusion strategy. Simultaneously, Branch 4 performs the zeroing operation on unimportant and anomalous time–frequency regions through the shared thresholding strategy. The combination of these strategies yields discriminative critical feature vectors, as illustrated in Figure 6c. In scenarios with a small data bias and severe imbalance, the Accuracy, F1-Score, and G-mean reach 99.47%, 99.47%, and 99.49%, respectively, which are all improved by 2% compared with those of Method 2. This further validates that the DSA-DRSB can extract rich, discriminative feature vectors even from a limited number of Samples, resulting in outstanding diagnostic performance.

**Figure 7:**
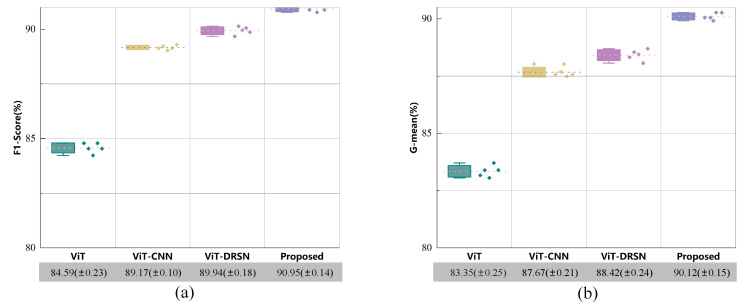
Diagnostic results of models in Dataset D.

The ablation experiment depicted in Figure 6 corroborates the rationale for incorporating convolutional layers into the Vision Transformer (ViT) architecture by comparing Method 4 to Method 2. However, it does not elucidate the extent to which this innovation surpasses the traditional CNN and the original DRSN. Moreover, to more effectively demonstrate the modeling capability and robustness of the DSA-DRSB on small Datasets, we conducted ablation experiments under the small and extremely imbalanced Dataset D with mixed loads, and the results are shown in Figure 7a,b, which show that ViT and ViT-CNN did not manage to achieve satisfactory results on severely imbalanced Datasets with mixed loads, with the F1-Score only reaching 84.59% and 89.17%. In contrast, ViT-DRSN introduces the DRSN after Embedding, which constrains the input feature vector solely through the utilization of Branch 1 (Average Pooling and channel thresholding). This removal of the non-critical parts of the signal enhances the model’s focus on vectors with crucial features, leading to strong performance on complex data. However, its F1-Score and G-mean never exceed 90%. In contrast, the proposed method achieves diagnostic results with both the F1-Score and G-mean surpassing 90% for the first time, reaching 90.95% and 90.12%, respectively. This further demonstrates that the method can effectively extract key discriminative features hidden in the signal, even in complex mixed loads, through the fusion thresholding and shared thresholding strategies, showcasing excellent modeling and anti-interference capabilities.

**Figure 8:**
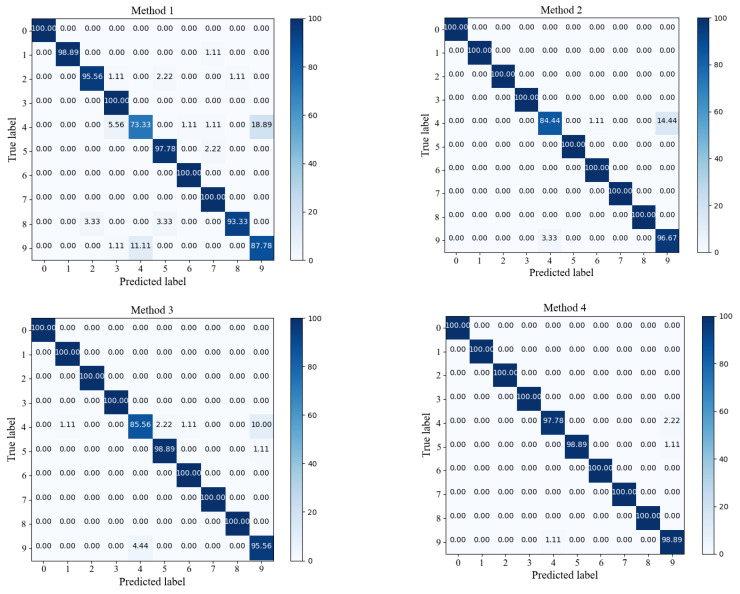
Confusion matrix of various methods (Dataset C).

To provide a clearer view of each model’s diagnostic results for each Class in the extremely imbalanced Dataset C, the confusion matrix visualizations for the first run of each model were generated. In these visualizations, the row labels represent the diagnostic results, while the column labels represent the true labels for fault Classes. As shown in Figure 8, the overall accuracies for the four models are as follows: 94.31% for Method 1, 97.20% for Method 2, 97.88% for Method 3, and 99.47% for Method 4. It can be observed that Method 1 did not achieve the expected performance for label 4. Nevertheless, Method 2, when enhanced with the IIRL, conducts additional in-depth feature extraction on challenging Samples belonging to this category, mitigating the CE’s uniform treatment of all Samples. As a result, the diagnostic Accuracy increases from 74.44% to 85.56%. Furthermore, with the addition of the DSA-DRSB, which has strong capabilities for modeling fine-grained features, the model’s diagnostic results improved from 85.56% to 97.78%. The main reason for this improvement is that the DSA-DRSB models local details of the input Samples, filters out irrelevant features, mitigates training biases, and makes the decision boundaries between Classes clearer, thereby enhancing the model’s Classification capabilities for each type of data.

**Figure 9:**
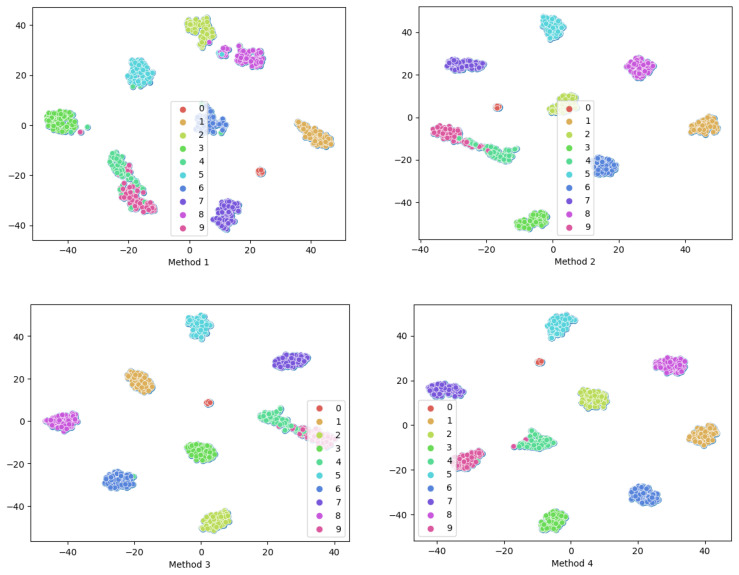
t-SNE feature visualization of different methods.

In addition to the previously mentioned visualization techniques involving confusion matrices, t-Distributed Stochastic Neighbor Embedding (t-SNE) is another commonly employed method for visualization. Continuing to use Dataset C as an example, the data were subjected to feature dimensionality reduction to obtain visualization results. As shown in Figure 9, in Method 1, there is significant overlap between different faults, such as faults 4 and 9, which lack clear decision boundaries. Similar situations are observed in Method 2 and Method 3, indicating insufficient fault-detection capabilities. However, the proposed methods are able to completely distinguish each fault Class (with only four Samples unable to be distinguished). There are clear distribution boundaries, enhancing the separability between different Classes and improving the model’s fault diagnostic capabilities.

#### 4.2.4. Comparison of Various Class Imbalanced Methods

**Figure 10:**
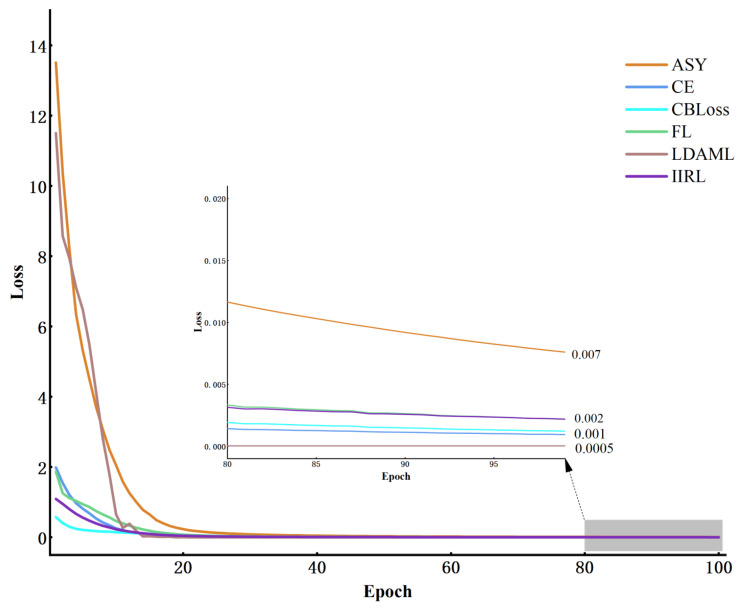
The convergence of different Loss methods on Dataset C.

To explore the effectiveness of the IIRL even further, we compared it to five currently available methods. This includes the CE, which achieves excellent results on Balanced Datasets, and four techniques that perform well on long-tailed Datasets: Class Balanced (CB), Asymmetric Loss (ASY) [37], LDAM Loss (LDAML), and Focal Loss (FL) methods, all with their best parameter settings as specified in the respective papers. The experiments were performed on Dataset A and Dataset C, and the diagnostic results are presented in Table 4. Whether the performance of the CE can be fully realized in the Balanced Dataset depends, to some extent, on the extent to which the feature vectors are captured. Undeniably, the performance of the CE with the DSA-DRSB is significantly better than other methods, proving once again that the DSA-DRSB with threshold fusion and shared thresholding has the ability to capture discriminative features in small Samples and imbalanced Datasets. The performance of the CB Loss inside the Balanced Dataset decreased compared to the CE [38], both in terms of the F1-Score and G-mean advantages. Additionally, the convergence curves for each Loss function on Dataset C are shown in Figure 10, with the diagnostic performance jointly reflected in Table 4 and Figure 10. It is evident that, when facing the challenges of long-tailed data, all methods except the proposed ones are at risk of overfitting. For instance, the LDAML converges to a Loss value around 0.0005, but as shown in Table 4, its F1-Score and G-mean on Dataset C are only 94.35% and 93.95%, respectively, which is disappointing compared to the proposed methods’ F1-Score and G-mean of 99.47% and 99.50%. Additionally, inspection of Figure 10 reveals that the advocated method consistently preserves convergence smoothness throughout the model training phase, without exhibiting pronounced oscillations akin to those observed in functions such as the LDAML and FL. Furthermore, although the CB Loss converges more uniformly than the IIRL, its F1-Score and G-mean metrics on the severely imbalanced Dataset C are merely 95.88% and 95.65%, respectively, as indicated in Table 4. This represents a considerable discrepancy from the IIRL’s respective scores of 99.47% and 99.50%, owing to the IIRL’s augmentation of the total Loss with Continuous incorporation of Loss about the challenging Samples within the Class. The persistent injection of this segment of Loss into the aggregate prevents the emergence of overfitting and facilitates stable convergence by iteratively grasping the elusive, highly discriminative features. This suggests that the IIRL holds promise in handling long-tailed distribution data.

Similarly, on Class-Balanced Datasets, the proposed methods achieved satisfactory results in terms of Accuracy compared to the CE. In summary, the IIRL can serve as a more-versatile alternative to the CE when facing both Balanced and imbalanced challenges.

### 4.3. Bearing Dataset from Laboratory

#### 4.3.1. Data Descriptions

**Figure 11:**
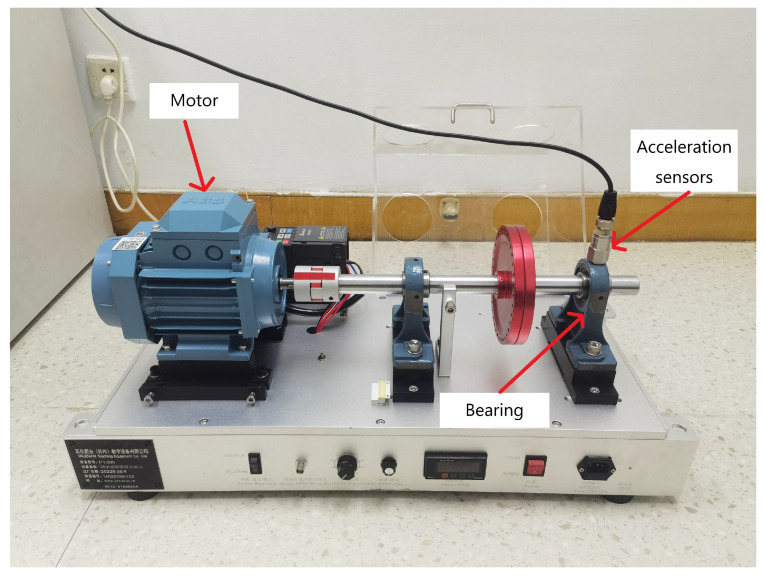
Laboratory Rolling bearing test bench.

In this case, a Rolling bearing device from the Laboratory was introduced as shown in Figure 11, which consists of components such as a motor, acceleration sensors, and support bearing. The acquisition frequency was 5K Hz, and the sensor device was placed in the 12 o’clock direction of the bearing for diagnostic signal acquisition. A total of four different load conditions (0-3HP) were collected, and the rotational speeds were 950 r/min, 1000 r/min, 1050 r/min, and 1100 r/min. Four major types of data were collected under each load, namely Normal, Rolling element failure, Inner Ring failure, and Outer Ring failure, in which each type of failure corresponds to three different damage diameters, which were 0.2, 0.3, and 0.4 mm, respectively. Taking the speed of the 950 r/m bearing state for example, they represent Light Inner Race fault (LIR), Middle Inner Race fault (MIR), Serious Inner Race fault (SIR), Light Outer Race fault (LOR), Middle Outer Race fault (MOR), Serious Outer Ring fault (SOR), Light Rolling Ball fault (LRB), Middle Rolling Ball fault (MRB) and Serious Rolling Ball fault (SRB). Therefore, there are a total of 10 Classes of data, one being Normal (N) with the other 9 representing different types of faults. Similarly, the original one-dimensional vibration signals were decomposed and transformed using the CWT. Taking the example of the bearings at 950 r/m, the signals for different fault locations are shown in Table 5. The construction of the Dataset still addresses the challenges of small and imbalanced cases, as detailed in Table 6, where Dataset D is a mixed-load small Sample severely imbalanced Dataset with a proportional mix of four loads.

#### 4.3.2. Ablation Experiment

**Figure 12:**
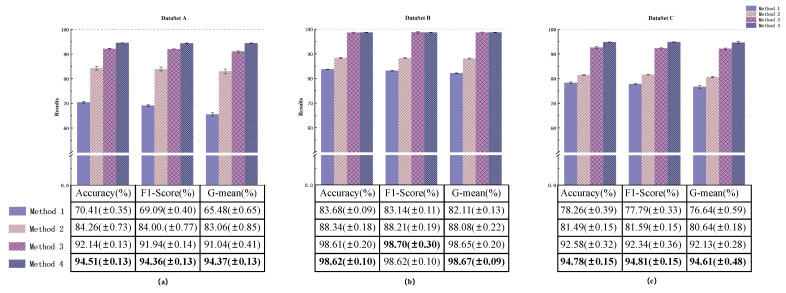
Diagnostic results of the models in Dataset A (**a**), Dataset B (**b**), and Dataset C (**c**).

**Figure 13:**
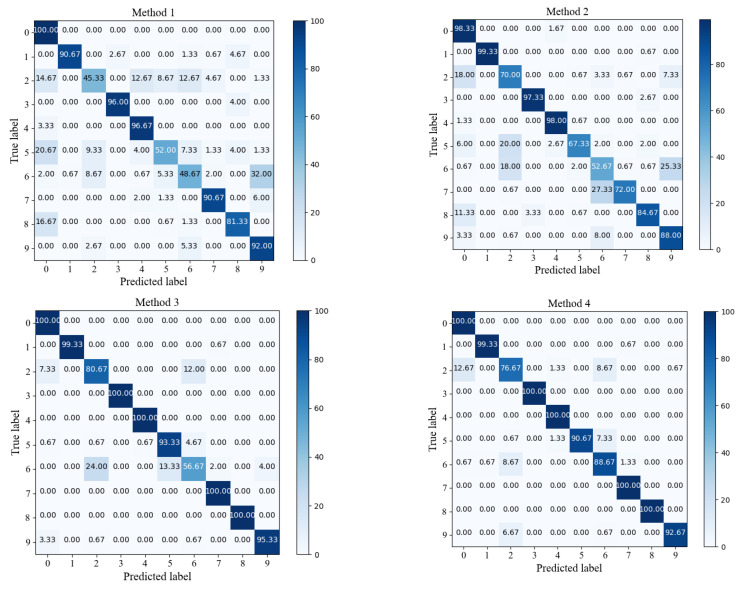
Confusion matrix of each method (Dataset C).

**Figure 14:**
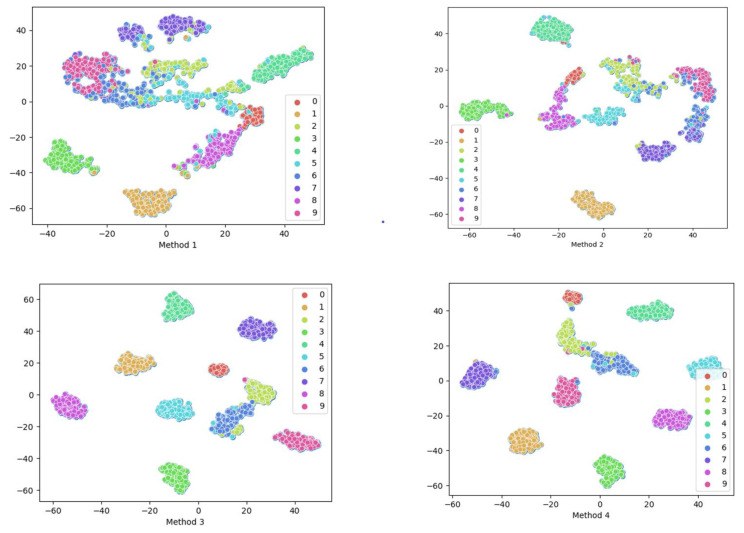
Feature visualization of each method via t-SNE.

For similar fault diagnosis tasks, the experimental parameters for Case 2 were kept consistent with Case 1, and the experimental results are shown in Figure 12. Through the evaluation and analysis of the proposed model using three metrics (Accuracy, F1-Score, G-mean), the experimental results indicate that the proposed method performs well under conditions of small Sample and Class imbalanced. Taking the highly imbalanced Dataset C as an example, the confusion matrices for each model’s first run are shown in Figure 13. The overall Classification Accuracy for the four models is 78.26%, 81.49%, 92.58%, and 94.78%. In the comparison between Method 2 and Method 3, it is evident that the proposed DSA-DRSB effectively learns crucial fault features even from a limited number of Samples. Furthermore, comparing Method 1 and Method 2, it is clear that the pure ViT model without the IIRL performs poorly on this Dataset, as it cannot assign different levels of attention to different Classes and cannot effectively mine difficult Samples within Classes. However, with the rebalancing effect of the IIRL, the probability of misidentifying fault Classes is significantly reduced. This pattern is also evident in the comparison between Method 3 and Method 4. The t-SNE feature visualization in Figure 14 provides a clearer demonstration of how the proposed model learns more-distinct decision boundaries, makes Classes more clustered within themselves, and separates them further from each other. These results once again highlight the promising application prospects of the proposed method in fault diagnosis under Class imbalance conditions.

Similarly, we conducted ablation experiments on the highly imbalanced Dataset D, which consists of small Samples under mixed loads collected under real operating conditions. The results are shown in Table 7. On the severely imbalanced Dataset with mixed loads, comparing ViT with ViT-CNN reveals that introducing convolutional layers into ViT significantly enhances the model’s ability to fit the data. When comparing ViT-CNN with ViT-DRSN, it is clear that the DRSN, by relying on soft thresholding to remove irrelevant redundant data, easily learns key features hidden in complex data. However, DRSN’s Average Pooling carries the risk of smoothing key time–frequency bands, making it challenging to capture critical time–frequency information hidden in the signal. What is gratifying is that our proposed method achieved diagnostic results exceeding 90% for both F1-Score and G-mean, reaching 91.03% and 90.50%, respectively. This further demonstrates that the proposed method can Balance Global and local fault time–frequency bands, providing excellent modeling and interference resistance capabilities.

#### 4.3.3. Comparison with Other Diagnosis Framework

**Figure 15:**
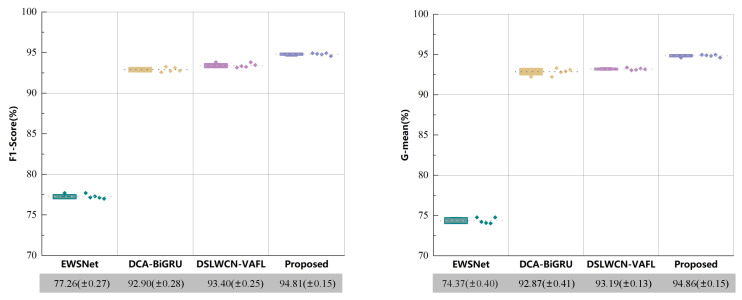
Comparison between IIRL and other imbalanced Classification frameworks.

To validate the superiority of the intelligent fault diagnosis method proposed in this paper, we performed comparative experiments with other recent models, such as EWSNet [39], DCA-BiGRU [34], and DSLWCN-VAFL [35]. To maintain a standard of impartiality, it is crucial to emphasize that the hyperparameters associated with the models discussed are harmonized with those adopted in the methodology presented within this paper. Figure 15 displays the results of comparing imbalanced Datasets at various scales. In the case of severe Class imbalance, EWSNet fails to achieve satisfactory results. The relatively better performance of the DCA-BiGRU and DSLWCN-VAFL depends on the successful training of these two models, which involve complex recurrent or convolutional architectures to handle long-term dependencies effectively. Additionally, as they aim to extract deeper features from limited Samples, they often face a shift in the training direction accompanied by the stacking of Network layers. In contrast, the Proposed method can extract deep Sample features with a limited number of Network layers, indicating a promising application scenario.

## 5. Conclusions

We introduced a Neural Network framework called DSADRSViT-IIRL to address the problem of small and imbalanced Datasets. To the best of our knowledge, this is the first implementation of combining the DRSN with ViT and proposing the DSA-DRSB. The ViT equipped with the DBA-DRSN blocks can identify local critical time–frequency bands that impact model Classification while emphasizing the Global fault information. We explored the superiority of the DSADRSViT-IIRL algorithm over other methods on two different bearing Datasets. In particular, the strategy of combining fusion thresholds and shared thresholds was used to alleviate the training pressure caused by redundant information and, at the same time, mining potential local key feature vectors, which enables the model to capture discriminative features in extremely limited Samples. Furthermore, we designed a novel cost Loss, IIRL, to address severe Class imbalance, rebalancing the contributions of both Interclass and Intraclass Samples to model convergence. Through comparative experiments, we found that the compared methods exhibited overfitting issues, further demonstrating the practicality of the IIRL. Finally, through ablation experiments, we validated the effectiveness of each module in the proposed method. Additionally, recent fault diagnosis methods were compared to demonstrate the viability of the proposed approach. The DSADRSViT-IIRL can effectively handle extremely limited and severely imbalanced signal Samples, aligning with the demands of modern industrial production and offering promising applications.

For future developments, lightweight architectures are worth exploring. Additionally, the DSADRSViT-IIRL may struggle to handle variable-speed Datasets. In the future, meta-learning, transfer learning, or reinforcement learning, when combined with ViT or other structures, can be employed to tackle more-complex variable-speed bearing data scenarios. 

## Figures and Tables

**Table 1:** Network parameters of the DSADRSViT-IIRL.

Name of Layers	Number	(Kernels, Strides, Padding)	Output
Embedding	1	-	(−1, 65, 64)
Conv	2	(3, 1, 1)	(−1, 65, 64)
GAP	1	-	(−1, 65, 1)
GMP	2	-	(−1, 65, 1)
Conv_Branch 1	2	(1, 1, 0)	(−1, 65)
Conv_Branch 2	2	(3, 3, 2)	(−1, 65)
Conv_Branch 4	2	(1, 1, 0)	(−1, 65)
Transformer Encoder	1	-	(−1, 65, 64)
MLP	1	-	(−1, 65, 64)
CLS	1	-	10

**Table 2:** Sample status of Rolling bearing fault Datasets.

Type of Fault	Motor Speed	Fault Diameter	TFRs
N	1750 rmp	-	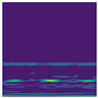
IR	1750 rmp	0.54 mm	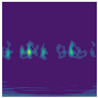
OR	1750 rmp	0.54 mm	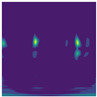
BF	1750 rmp	0.54 mm	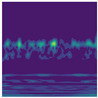

**Table 3:** Detailed description of Laboratory bearing Dataset.

Dataset	Number of Samples									
**Label**	**0**	**1**	**2**	**3**	**4**	**5**	**6**	**7**	**8**	**9**
**Type**	**N**	**SIR**	**MIR**	**CIR**	**SOR**	**MOR**	**COR**	**SRB**	**MRB**	**CRB**
A	9	9	9	9	9	9	9	9	9	9
B	90	20	20	20	20	20	20	20	20	20
C	90	9	9	9	9	9	9	9	9	9
D	90	9	9	9	9	9	9	9	9	9

**Table 4:** Comparison of Accuracy of different Loss methods.

Loss	F1-Score (%)		G-Mean (%)	
	Dataset A	Dataset C	Dataset A	Dataset C
CE	**99.44 (±0.11)**	95.69 (±0.07)	**99.40 (±0.11)**	95.59 (±0.08)
CB	96.79 (±0.09)	95.88 (±0.15)	96.54 (±0.11)	95.65 (±0.18)
ASY	98.35 (±0.48)	96.21 (±0.07)	98.30 (±0.50)	96.28 (±0.07)
LDAML	95.97 (±0.07)	94.35 (±0.08)	95.55 (±0.07)	93.95 (±0.10)
FL	97.44 (±0.25)	98.75 (±0.26)	97.26 (±0.30)	98.81 (±0.25)
proposed	99.39 (±0.14)	**99.47 (±0.07)**	99.38 (±0.15)	**99.50 (±0.07)**

**Table 5:** Sample status of Laboratory fault Datasets.

Type of Fault	Motor Speed	Fault Position	TFRs
N	950 r/m	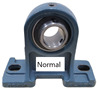	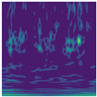
IR	950 r/m	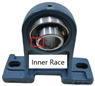	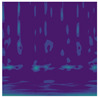
OR	950 r/m	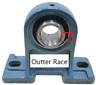	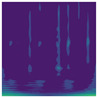
BF	950 r/m	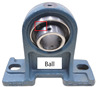	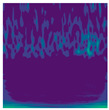

**Table 6:** Detailed description of Laboratory bearing Dataset.

Dataset	Number of Samples									
**Label**	**0**	**1**	**2**	**3**	**4**	**5**	**6**	**7**	**8**	**9**
**Type**	**N**	**LIR**	**MIR**	**SIR**	**LOR**	**MOR**	**SOR**	**LRB**	**MRB**	**SRB**
A	9	9	9	9	9	9	9	9	9	9
B	90	20	20	20	20	20	20	20	20	20
C	90	9	9	9	9	9	9	9	9	9
D	90	9	9	9	9	9	9	9	9	9

**Table 7:** Diagnostic results of models in Dataset D.

Results	Dataset D		
	Accuracy (%)	F1-Score (%)	G-Mean (%)
ViT	71.00 (±0.23)	70.34 (±0.22)	68.29 (±0.26)
ViT-CNN	82.88 (±0.50)	82.39 (±0.50)	79.87 (±0.31)
ViT-DRSN	87.18 (±0.13)	87.22 (±0.12)	85.14 (±0.16)
proposed	**91.14 (±0.11)**	**91.03 (±0.13)**	**90.50 (±0.46)**

## Data Availability

The data used in this study can be requested from the corresponding author. Due to confidentiality requirements in the Laboratory where the testing equipment is located, these data are not publicly disclosed.

## References

[1] He C., Shi H., Li J. (2023). IDSN: A one-stage interpretable and differentiable STFT domain adaptation Network for traction motor of high-speed trains cross-machine diagnosis. Mech. Syst. Signal Process..

[2] Liu Y., Jiang H., Liu C., Y W., S W. (2022). Data-augmented Wavelet capsule generative adversarial Network for Rolling bearing fault diagnosis. Knowl. Based Syst..

[3] Zou L., Zhuang K., Zhou A., Hu J. (2023). Bayesian optimization and channel-fusion-based convolutional autoencoder Network for fault diagnosis of rotating machinery. Eng. Struct..

[4] Wang Z., Yang J., Guo Y., Hu J. (2022). Unknown fault feature extraction of Rolling bearings under variable speed conditions based on statistical complexity measures. Mech. Syst. Signal. Process..

[5] Liang P., Wang W., Yuan X., Liu S., Zhang L., Cheng Y. (2022). Intelligent fault diagnosis of Rolling bearing based on Wavelet transform and improved ResNet under noisy labels and environment. Eng. Appl. Artif. Intell..

[6] Tong J., Tang S., Wu Y., Pan H., Zheng J. (2023). A fault diagnosis method of Rolling bearing based on improved Deep Residual Shrinkage Networks. Measurement.

[7] Zhang T., Chen J., Li F., Zhang K., Lv H., He S., Xu E. (2022). Intelligent fault diagnosis of machines with small imbalanced data: A state-of-the-art review and possible extensions. ISA Trans..

[8] Yang J., Xie G., Yang Y. (2020). An improved ensemble fusion autoencoder model for fault diagnosis from imbalanced and incomplete data. Control. Eng. Pract..

[9] Wu H., Triebe M.J., Sutherland J.W. (2023). A transformer-based approach for novel fault detection and fault Classification/diagnosis in manufacturing: A rotary system application. J. Manuf. Syst..

[10] Jia L., Chow T.W.S., Wang Y., Yuan Y. (2022). Multiscale Residual Attention Convolutional Neural Network for Bearing Fault Diagnosis. IEEE Trans. Instrum. Meas..

[11] Huang Y., Liao A., Hu D., Shi W., Zheng S. (2022). Multi-scale convolutional Network with channel attention mechanism for Rolling bearing fault diagnosis. Measurement.

[12] Dosovitskiy A., Beyer L., Kolesnikov A. (2020). An image is Worth 16x16 Words: Transformers for Image Recognition at Scale. arXiv.

[13] Tang X., Xu Z., Wang Z. (2022). A Novel Fault Diagnosis Method of Rolling Bearing Based on Integrated Vision Transformer Model. Sensors.

[14] Zhou K., Tong Y., Li X., Wei X., Huang H., Song K., Chen X. (2023). Exploring Global attention mechanism on fault detection and diagnosis for complex engineering processes. Process. Saf. Environ. Prot..

[15] Zhang Q., Yang Y. (2021). ResT: An Efficient Transformer for Visual Recognition. arXiv.

[16] Wu H., Xiao B., Codella N., Liu M., Dai X., Yuan L., Zhang L. (2021). CvT: Introducing Convolutions to Vision Transformers. arXiv.

[17] Tian A., Zhang Y., Ma C., Chen H., Sheng W., Zhou S. (2023). Noise-robust machinery fault diagnosis based on self-attention mechanism in Wavelet domain. Measurement.

[18] Pei X., Dong S., Tang B., Pan X., Sheng W., Zhou S. (2022). Bearing Running State Recognition Method Based on Feature-to-Noise Energy Ratio and Improved Deep Residual Shrinkage Network. IEEE ASME Trans. Mechatron..

[19] Chen Z., Wu K., Wu J., Deng C., Wang Y. (2023). Residual shrinkage transformer relation Network for intelligent fault detection of industrial robot with zero-fault Samples. Knowl. Based Syst..

[20] Cao K., Wei C., Gaidon A., Arechiga N., Ma T. (2019). Learning ImBalanced Datasets with Label-Distribution-Aware Margin Loss. arXiv.

[21] Cui Y., Jia M., Lin T., Song Y., Serge B. (2019). Class-Balanced Loss Based on Effective Number of Samples. arXiv.

[22] Lin T., Goyal P., Girshick R., He K., Dollár P. Focal Loss for dense object detection. Proceedings of the IEEE International Conference on Computer Vision.

[23] Zhao X., Yao J., Deng W., Jia M., Zheng L. (2022). Normalized Conditional Variational Auto-Encoder with adaptive Focal Loss for imbalanced fault diagnosis of Bearing-Rotor system. Mech. Syst. Signal. Process..

[24] Xiao J., Li C., Liu B., Huang J., Xie L. (2022). Prediction of wind turbine blade icing fault based on selective Deep Ensemble model. Knowl. Based Syst..

[25] Xia M., Li T., Xu L., Liu L., Silva C. (2018). Fault Diagnosis for Rotating Machinery Using Multiple Sensors and Convolutional Neural Networks. IEEE ASME Trans. Mechatron..

[26] Zhao M., Zhong S., Fu X., Tang B., Pecht M. (2020). Deep Residual Shrinkage Networks for Fault Diagnosis. IEEE Trans. Ind. Inform..

[27] Pang X., Xue X., Jiang W., Lu K. (2021). An Investigation Into Fault Diagnosis of Planetary Gearboxes Using A Bispectrum Convolutional Neural Network. IEEE ASME Trans. Mechatron..

[28] Li Y., Zhang K., Cao J., Timofte R., Gool L. (2021). LocalViT: Bringing Locality to Vision Transformers. arXiv.

[29] Zhang S., Liu Z., Chen Y., Jin Y., Bai G. (2023). Selective kernel convolution deep residual Network based on channel-spatial attention mechanism and feature fusion for mechanical fault diagnosis. ISA Trans..

[30] Chen B., Liu T., He C., Liu Z., Zhang L. (2022). Fault Diagnosis for Limited Annotation Signals and Strong Noise Based on Interpretable Attention Mechanism. IEEE Sens. J..

[31] He K., Zhang X., Ren S., Jian S. Deep Residual Learning for Image Recognition. Proceedings of the IEEE Conference on Computer Vision and Pattern Recognition (CVPR).

[32] Ding X., Zhang X., Ma N., Han J., Ding G., Sun J. RepVGG: Making VGG-style ConvNets Great Again. Proceedings of the IEEE Conference on Computer Vision and Pattern Recognition (CVPR).

[33] Xu Z., Tang X., Wang Z. (2023). A Multi-Information Fusion ViT Model and Its Application to the Fault Diagnosis of Bearing with Small Data Samples. Machines.

[34] Zhang X., He C., Lu Y., Chen B., Zhu L., Zhang L. (2022). Fault diagnosis for small Samples based on attention mechanism. Measurement.

[35] Chen B., Zhang L., Liu T., Li H., He C. (2022). Lightweight Network with Variable Asymmetric Rebalancing Strategy for Small and ImBalanced Fault Diagnosis. Machines.

[36] Shrivastava A., Gupta A., Girshick R. (2016). Training Regionbased Object Detectors with Online Hard Example Mining. arXiv.

[37] Ben-Baruch E., Ridnik T., Zamir N., Noy A., Friedman I., Protter P., Zelnik-Manor L. (2022). Asymmetric Loss For Multi-Label Classification. arXiv.

[38] Smith L. (2022). Cyclical Focal Loss. arXiv.

[39] He C., Shi H., Si J., Li J. (2023). Physics-informed interpretable Wavelet weight initialization and Balanced dynamic adaptive threshold for intelligent fault diagnosis of Rolling bearings. J. Manuf. Syst..

